# Neuro Nonsense

**DOI:** 10.1371/journal.pbio.1001005

**Published:** 2010-12-14

**Authors:** Ben A. Barres

**Affiliations:** Department of Neurobiology, Stanford University School of Medicine, Stanford, California, United States of America

**Figure:**
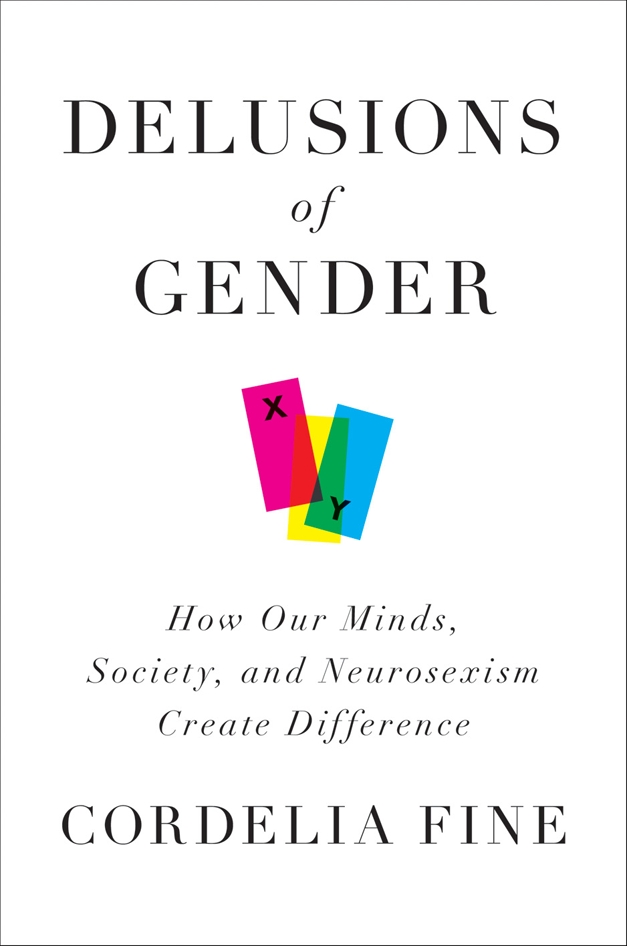
Fine C (2010) Delusions of Gender: How Our Minds, Society and Neurosexism Create Difference. New York: W. W. Norton and Company. 338p. ISBN 978-00393068382 (hardcover). US$25.95.

No one disputes that male and female brains are different or that males and females differ in their accomplishments. But are these two facts related? A few years ago Harvard President Larry Summers suggested that the answer is yes. He proposed that innate brain differences help to account for the dearth of successful women in science, provoking much heated debate. Reporters called it the story that would not die. Unlike most news stories that exhaust themselves after a few days, this story stayed in the news for months, and even years later continues to inspire debate. Apparently many of us think we already know the answer to this question—the subject of Cordelia Fine's highly readable and enjoyable new book *Delusions of Gender: How Our Minds, Society, and Neurosexism Create Difference* [1]. At least half of us—not just the men—seem to think the answer is yes whereas the other half say not so.

You all know where I stand on this issue. Based on my experiences as a neurobiologist and as a transgendered person, I have previously argued that innate sex differences in the brain are not relevant to real-world accomplishments [2],[3]. Without question, male and female brains have different circuits that help to control their different reproductive behaviors. So it has long seemed an easy step to believe that such anatomic changes also underlie supposed gender differences in cognitive abilities. Rather, in a theme that Fine elegantly expands on, it is *the idea itself* that women are innately less capable that may be the primary cause of differences in accomplishment. This idea Fine appropriately dubs “neurosexism.” This idea was long ago powerfully encapsulated in the concept of “stereotype threat,” the phenomenon in which members of a sex or race perform substantially worse on a test—and perhaps in real-world environments—when they are led to believe before the test that they are innately less capable [4].

Fine is an academic psychologist who previously authored *A Mind of Its Own: How Your Brain Distorts and Deceives* [5]. She decided to write *Delusions of Gender* after she discovered that her young son was taught at school that boys were not as good at empathizing as girls. Stunned by this experience, Fine critically scoured the relevant scientific literature. Her analysis of this data should be required reading for every neurobiology student, if not every human being. (I wonder if Norton Press might be so good as to send Larry Summers a free copy?) The main theme of Fine's new book is that current widespread beliefs about gender—that is, we needn't worry about social or cultural factors leading to sex inequality because hardwired differences between the sexes are to blame—just don't bear up to scrutiny.

For instance, many studies have found that developmental differences in testosterone level result in permanent differences in brain hard-wiring. Are these differences relevant to cognitive abilities? Simon Baron-Cohen thinks so, reporting that developmental hormonal differences cause men to be more systematic thinkers, better at analyzing and exploring, while making women more empathetic [6].His findings have been used by many, including Steven Pinker [7] and Peter Lawrence [8] to argue that men are innately more likely to succeed in science. But Fine raises devastating questions about Baron-Cohen's methodology, raising serious concerns about poorly defined and socially biased questions used in his questionnaires in some of these studies. In a highly influential study, for instance, he and a student reported that newborn boys prefer to look at mobiles, whereas newborn girls prefer to look at faces. But the study's design did not prevent the possibility that the experimenter might inadvertently give different cues to the boys and girls. Her critique raises significant doubt about whether Baron-Cohen's conclusions about sex differences are correct. Even if his measurements were correct, it is not clear that differences observed in newborns are relevant to adult behavior. Lastly, the differences observed were not particularly large, a problem that, Fine points out, applies to many other reported cognitive differences as well. Such differences are often small enough that social experiences can often easily remove them. For instance, the small gender differences observed in spatial abilities can largely be obliterated by practicing mechanical tasks.

Fine raises many points that are often neglected in interpreting the results of studies on cognitive sex differences. For instance, she points out that only studies that find a difference are published whereas negative results, which may be more common, are not reported. She points out the many interpretation abuses of modern neuroimaging studies. For instance, the brain's well-documented plasticity means that one cannot interpret the mere presence of an imaging difference as evidence of innate disparity. Fine also discusses abuses of statistical analyses. Often the studies that report sex differences have surprisingly small sample sizes. It is refreshing to read such a critical analysis of this literature, which is lacking in so many of the previous writings on gender differences. Readers of this book may also enjoy another recently published book by Rebecca Jordan-Young called *Brain Storm:The Flaws in the Sciences of Sex Differences* [9], which also critically examines the evidence that cognitive sex differences are hardwired into the brain.

Importantly, Fine points out how much writing about sex differences consists of just-so stories that can be easily constructed because the relationship between brain structure and cognitive function is still poorly understood. Such just-so stories are also the bread and butter of a field known as evolutionary psychology. Darwin, Pinker [7], and others have long argued that men have evolved different neural circuits that imbue them with different (superior) cognitive abilities that favor more competitive and risk-taking behaviors. But the field of evolutionary psychology has been the subject of many recent critiques. Not only are its hypotheses untestable and unfalsifiable, they also involve circular reasoning; the thinking starts out with sexist Darwinian biases, like males are more competitive, and then ends at the same starting point, concluding that male neural circuits have evolved for competition.

Women scientists have long argued against this idea. In an essay called “The Woman that Never Evolved” (1981), Sarah Blaffer Hrdy argued that Darwin's notion of a passive female role in sexual selection stemmed from Victorian social conventions. In a piece called “The Evolution of Woman: An Inquiry Into the Dogma of Her Inferiority,” Eliza Burt Gamble (1894) argued that where Darwin interpreted his data to indicate that men were superior to women, she saw female superiority. And perhaps most importantly in “The Politics of Women's Biology,” Ruth Hubbard (1990) concluded that so long as biology as an enterprise is almost exclusively a male occupation, it will be a biased science, masquerading as objective, and will make unfounded claims about women's biology that will justify the inferior status of women.

Surprisingly, as Dr. Fine points out, women are also prominent contributors to the neurosexist literature. Her detailed analysis of *The Female Brain* by Dr.Louann Brizendine [10] is remarkable in showing the large number of serious flaws and errors that this book contains. As one example, Fine tracked down every neuroscience study that Brizendine claimed showed that the female brain was wired to emphathize and found frequent deployment of misleading practices in discussing these studies. In one study reporting that therapists develop good rapport with their clients by mirroring their actions, Brizendine reports that all of the therapists who showed these responses happened to be women, but she failed to mention that only female therapists were used in the study. Brizendane also claimed that a meta-analysis found female superiority in decoding nonverbal emotional expressions, but in fact this meta-analysis concluded there were only minor differences between girls (54%) and boys (46%).

Finally, Fine supplies some wonderful pointers to those who write books about gender: Do not suggest that parents or teachers treat boys and girls differently because of differences observed in their brains. Exercise extreme caution when making the perilous leap from brain structure to psychological function. Most importantly, please don't make stuff up! I think that Fine is at her best when she points out that neuroscientists have responsibility for how their findings are interpreted. They—and reviewers and editors—bear a heavier burden of caution because of the social implications of this work. She concludes that neurosexism promotes damaging, limiting, and potentially self-fulfilling stereotypes, powerfully reminding us that neuroscience can be dangerous when mishandled.
